# Conditional Hfq Association with Small Noncoding RNAs in Pseudomonas aeruginosa Revealed through Comparative UV Cross-Linking Immunoprecipitation Followed by High-Throughput Sequencing

**DOI:** 10.1128/mSystems.00590-19

**Published:** 2019-12-03

**Authors:** Kotaro Chihara, Thorsten Bischler, Lars Barquist, Vivian A. Monzon, Naohiro Noda, Jörg Vogel, Satoshi Tsuneda

**Affiliations:** a Department of Life Science and Medical Bioscience, Waseda University, Tokyo, Japan; b Biomedical Research Institute, National Institute of Advanced Industrial Science and Technology, Ibaraki, Japan; c Core Unit Systems Medicine, University Hospital of Würzburg, Würzburg, Germany; d Helmholtz Institute for RNA-based Infection Research, Helmholtz Center for Infection Research, Würzburg, Germany; e Faculty of Medicine, University of Würzburg, Würzburg, Germany; f European Molecular Biology Laboratory, European Bioinformatics Institute, Hinxton, United Kingdom; g Institute of Molecular Infection Biology, University of Würzburg, Würzburg, Germany; University of Wisconsin-Madison

**Keywords:** CLIP-seq, Hfq, *Pseudomonas aeruginosa*, biofilms, noncoding RNA

## Abstract

Bacterial small noncoding RNAs (sRNAs) play posttranscriptional regulatory roles in cellular responses to changing environmental cues and in adaptation to harsh conditions. Generally, the RNA-binding protein Hfq helps sRNAs associate with target mRNAs to modulate their translation and to modify global RNA pools depending on physiological state. Here, a combination of *in vivo* UV cross-linking immunoprecipitation followed by high-throughput sequencing (CLIP-seq) and total RNA-seq showed that Hfq interacts with different regions of the Pseudomonas aeruginosa transcriptome under planktonic versus biofilm conditions. In the present approach, P. aeruginosa Hfq preferentially interacted with repeats of the AAN triplet motif at mRNA 5′ untranslated regions (UTRs) and sRNAs and U-rich sequences at rho-independent terminators. Further transcriptome analysis suggested that the association of sRNAs with Hfq is primarily a function of their expression levels, strongly supporting the notion that the pool of Hfq-associated RNAs is equilibrated by RNA concentration-driven cycling on and off Hfq. Overall, our combinatorial CLIP-seq and total RNA-seq approach highlights conditional sRNA associations with Hfq as a novel aspect of posttranscriptional regulation in P. aeruginosa.

**IMPORTANCE** The Gram-negative bacterium P. aeruginosa is ubiquitously distributed in diverse environments and can cause severe biofilm-related infections in at-risk individuals. Although the presence of a large number of putative sRNAs and widely conserved RNA chaperones in this bacterium implies the importance of posttranscriptional regulatory networks for environmental fluctuations, limited information is available regarding the global role of RNA chaperones such as Hfq in the P. aeruginosa transcriptome, especially under different environmental conditions. Here, we characterize Hfq-dependent differences in gene expression and biological processes in two physiological states: the planktonic and biofilm forms. A combinatorial comparative CLIP-seq and total RNA-seq approach uncovered condition-dependent association of RNAs with Hfq *in vivo* and expands the potential direct regulatory targets of Hfq in the P. aeruginosa transcriptome.

## Abstract (precis)

The Gram-negative bacterium P. aeruginosa is ubiquitously distributed in diverse environments and can cause severe biofilm-related infections in at-risk individuals. Although the presence of a large number of putative sRNAs and widely conserved RNA chaperones in this bacterium implies the importance of posttranscriptional regulatory networks for environmental fluctuations, limited information is available regarding the global role of RNA chaperones such as Hfq in the P. aeruginosa transcriptome, especially under different environmental conditions. Here, we characterize Hfq-dependent differences in gene expression and biological processes in two physiological states: the planktonic and biofilm forms. A combinatorial comparative CLIP-seq and total RNA-seq approach uncovered condition-dependent association of RNAs with Hfq *in vivo* and expands the potential direct regulatory targets of Hfq in the P. aeruginosa transcriptome.

## INTRODUCTION

To thrive in fluctuating environments, bacteria must adapt to environmental threats such as temperature fluctuations, nutrient/oxygen limitations, and antibiotic exposure. In this context, regulatory noncoding RNAs (ncRNAs) have recently been implicated in posttranscriptional regulation of diverse cellular processes, including metabolism, stress response, and virulence (1). Among the ncRNAs, small noncoding RNAs (sRNAs) are transcribed distal to their target RNAs. Regulation is generally accomplished through incomplete base pair formation, owing to which sRNAs often need RNA-binding proteins (RBPs) to facilitate base pairing with their target RNAs (2). Hfq is one of the most extensively studied RBPs among Gram-negative bacteria (3). The primary mode of action of Hfq is through acceleration of sRNA-mRNA annealing (4, 5) and subsequent RNA stabilization or degradation (6–8) though alternative regulatory mechanisms have also been described (9–11).

Pseudomonas aeruginosa is a notorious bacterium as an opportunistic biofilm-forming pathogen of burn wounds, medical devices, and the lungs of immunocompromised individuals (12). This bacterium can grow in a wide range of environments other than the human body, its high adaptability potentially resulting from its large genome and unusually high proportion of transcriptional and posttranscriptional regulators (13–15). Although 680 ncRNAs have been putatively detected thus far in P. aeruginosa PAO1 and PA14 genomes (16–18), only a few sRNAs have been experimentally validated (19). For example, PrrF1/PrrF2 (PrrF1/2) are expressed in an iron acquisition regulatory factor Fur-dependent manner (20) and contribute to translational regulation of iron-dependent proteins, serving a similar functional role to the *Enterobacteriaceae* sRNA RyhB (21). P. aeruginosa expresses the RNA chaperone Hfq; however, its C-terminal domain (CTD) is truncated in comparison to CTDs of the model Hfq proteins of Escherichia coli and *Salmonella* (22). P. aeruginosa Hfq exerts pleiotropic effects: the production of virulence factors, quorum sensing, and motility are impaired in the Δ*hfq* strain (23, 24). Additionally, the catabolite repression control protein Crc represses the function involved in utilization of less preferred carbon sources, and the Hfq/Crc-binding sRNA CrcZ binds reciprocally and is cross-regulated with Hfq in P. aeruginosa (25–27). Recently, Kambara et al. have shown that Hfq binds hundreds of nascent transcripts cotranscriptionally, often in concert with Crc (28).

New technologies based on high-throughput sequencing are increasingly providing insight into the functions of RBPs and their associated sRNAs (29, 30). In particular, *in vivo* UV cross-linking immunoprecipitation followed by high-throughput sequencing (CLIP-seq) can detect transcriptome-wide binding partners of RBPs and identifies common binding sites down to single-nucleotide resolution (31–34). Moreover, *in vivo* preferential ligation of RNAs has begun to unravel the sRNA interactome (35–38). While these large-scale approaches have provided global insights into individual RBP-binding RNAs, the mechanism and functions of Hfq-mediated regulation of the P. aeruginosa transcriptome remain unclear, especially under different environmental conditions.

In this study, we performed simultaneous CLIP-seq (32, 34) and total RNA-seq to understand the molecular mode of action and physiological effects of Hfq under two medically and scientifically relevant conditions: planktonic and biofilm growth. Our comparative approach highlights competitive sRNA regulation depending on expression and allows us to reassess the key functions of Hfq in P. aeruginosa.

## RESULTS

### Identification of different RNA interactions between planktonic and biofilm forms.

**FIG 1:**
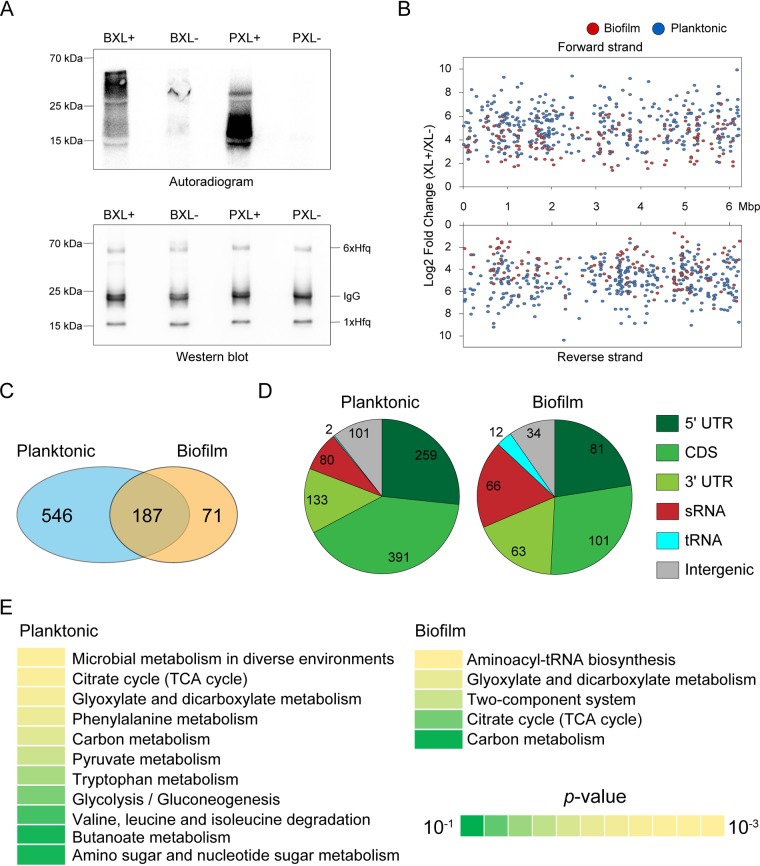
Overview of cross-linking immunoprecipitation with high-throughput sequencing (CLIP-seq) analysis of Pseudomonas aeruginosa Hfq under planktonic and biofilm conditions. (A) Autoradiogram and Western blotting of the CLIP-enriched Hfq-RNA complex under two physiological conditions. XL+, cross-linking; XL-, non-cross-linking; P, planktonic; B, biofilm. Biological replicates are shown in Fig. S1 in the supplemental material. (B) The distribution of Hfq peaks throughout the P. aeruginosa PAO1 genome. Peaks from planktonic and biofilm conditions are highlighted in blue and red, respectively. (C) The Venn diagram shows the number of detected peaks under planktonic and biofilm conditions. Two peaks from both conditions wherein both the start and stop positions are within 40 nt are regarded as the same peak. (D) Classification of Hfq peaks into RNA classes (5′ UTR, CDS, 3′ UTR, sRNA, tRNA, and intergenic peaks). The 5′ UTRs and 3′ UTRs were annotated from TSSs validated by differential RNA-seq (39) and terminators predicted by TransTermHP (40), as well as manual curation of sRNAs from size selection sRNA-seq conducted by previous researches (16, 17). (E) DAVID enrichment analysis of Hfq peaks from 502 and 180 mRNAs except for intergenic regions under planktonic and biofilm conditions, respectively. The results of KEGG pathway enrichment analysis are presented. Overall results are shown in Table S2.

To investigate the interactions of Hfq with target RNAs under two pervasive conditions, i.e., planktonic and biofilm forms, we employed comparative CLIP-seq of the P. aeruginosa PAO1 *hfq*::3×FLAG strain. In this strain, growth rate, colony morphology, and pigment production remained unimpaired (see Fig. S1 in the supplemental material). UV irradiation to induce RNA-protein cross-linking was carried out on early stationary planktonic cultures (optical density at 600 nm [OD_600_] of 2.0), and suspensions of colony biofilms formed on a cellulose membrane on Luria-Bertani (LB) agar for 48 h. Autoradiography and Western blot analyses indicated that UV cross-linking and anti-FLAG coimmunoprecipitation with stringent washing successfully enriched Hfq-RNA complex under both physiological conditions (Fig. 1A and Fig. S2).

10.1128/mSystems.00590-19.1 FIG S1 3×FLAG insertion downstream of *hfq* did not impair bacterial physiology. (A) Growth rate of wild-type, *hfq*::3×FLAG, and Δ*hfq* strains. (B) Representative figure of colony morphologies of wild-type, *hfq*::3×FLAG, and Δ*hfq* strains incubated on 1% tryptone agar with 20 μg/ml Coomassie brilliant blue and 40 μg/ml Congo red for 5 days at 25°C. (C) Overnight planktonic cultures grown in LB medium. Pigmentation was upregulated only in a Δ*hfq* strain. Download FIG S1, TIF file, 1.3 MB. Copyright © 2019 Chihara et al. 2019 Chihara et al. This content is distributed under the terms of the Creative Commons Attribution 4.0 International license.

10.1128/mSystems.00590-19.2 FIG S2 The Hfq-RNA complex was successfully detected in the Pseudomonas aeruginosa PAO1 *hfq*::3×FLAG strain under both planktonic and biofilm conditions. (A) Cell extracts from both the PAO1 wild-type and *hfq*::3×FLAG strain were confirmed via Western blot analysis using anti-FLAG antibody. Hfq::3×FLAG protein was only detected from PAO1 *hfq*::3×FLAG strain. Detection was carried out under planktonic (OD_600_ = 1.0, 2.0, and 3.0) and colony biofilm (24-h-old) conditions. (B) Autoradiogram and Western blots indicate that cross-linking immunoprecipitation enriched the Hfq-RNA complex in both physiological conditions in biological replicates, as shown in Fig. 1A. Download FIG S2, TIF file, 2.6 MB. Copyright © 2019 Chihara et al. 2019 Chihara et al. This content is distributed under the terms of the Creative Commons Attribution 4.0 International license.

We performed next-generation sequencing for both cross-linked and non-cross-linked samples in three biological replicates, subsequently seeing good correlations within each experimental condition (Fig. S3). Peak calling using the tool PEAKachu (https://github.com/tbischler/PEAKachu; see Materials and Methods) identified 991 putative Hfq-binding sites as peaks (average peak length ± standard deviation, 44.2 ± 14.9 nucleotides [nt]) with significant enrichment in cross-linked samples throughout the P. aeruginosa PAO1 genome (Fig. 1B; also Fig. S4 and Table S1). We identified 187 overlapping peaks between planktonic and biofilm conditions, where overlapping peaks were defined as having both the start and stop positions within 40 nt of each other (Fig. 1C). Significant peaks were classified on the basis of RNA classes (Fig. 1D). We generated an untranslated region (UTR) annotation in accordance with previously reported transcription start site (TSS) data from differential RNA-seq (39) and terminators predicted via TransTermHP (40) via the pipeline ANNOgesic (41), along with manual curation of sRNAs from size selection sRNA-seq conducted previously (16, 17). Under both conditions, the majority of the peaks were classified into mRNAs (5′ UTR, coding DNA sequence [CDS], and 3′ UTR) and sRNAs, with fewer peaks for remaining unannotated intergenic regions.

10.1128/mSystems.00590-19.3 FIG S3 Heat map of correlation coefficients upon cross-linking immunoprecipitation with high-throughput sequencing. Correlation coefficient ρ was calculated from cross-linking/non-cross-linking (XL+ or XL−), forward/reverse strands (F or R), planktonic/biofilm (P or B), and biological replicates (1 to 3). In both strands, ρ was low between cross-linking and non-cross-linking samples, implying that UV treatment effectively cross-linked the RNA-Hfq complex. Download FIG S3, TIF file, 1.7 MB. Copyright © 2019 Chihara et al. 2019 Chihara et al. This content is distributed under the terms of the Creative Commons Attribution 4.0 International license.

10.1128/mSystems.00590-19.4 FIG S4 The results of exploratory analysis. Frequency plots of matched cross-linked and background samples. The axes indicate read counts, and the colored regions indicate the frequency of each *x-y* pair. Download FIG S4, TIF file, 1.5 MB. Copyright © 2019 Chihara et al. 2019 Chihara et al. This content is distributed under the terms of the Creative Commons Attribution 4.0 International license.

10.1128/mSystems.00590-19.7 TABLE S1 Hfq peaks detected by CLIP-seq. Download Table S1, XLSX file, 0.2 MB. Copyright © 2019 Chihara et al. 2019 Chihara et al. This content is distributed under the terms of the Creative Commons Attribution 4.0 International license.

To determine the metabolic pathways in which Hfq-binding RNAs are enriched, DAVID enrichment analysis was performed for the peaks, with the exception of the sRNAs and intergenic regions, under planktonic and biofilm conditions with a modified Fisher’s exact *P* value threshold of <0.1 (Table S2) (42). In the planktonic growth, genes related to carbon metabolism, such as the tricarboxylic acid (TCA) cycle, glycolysis, and gluconeogenesis, were enriched, consistent with the interaction between carbon catabolite repression control protein Crc and Hfq (Fig. 1E, left) (27, 28). In contrast, genes related to carbon metabolism and two-component systems were enriched under the biofilm condition (Fig. 1E, right). Intriguingly, aminoacyl-tRNA biosynthesis was specifically highly enriched under the biofilm condition. Together, comparative CLIP-seq analysis between planktonic and biofilm forms identified different RNAs associated with Hfq and dynamic regulations of biological processes.

10.1128/mSystems.00590-19.8 TABLE S2 DAVID enrichment analysis data for genes identified by CLIP-seq. Download Table S2, XLSX file, 0.05 MB. Copyright © 2019 Chihara et al. 2019 Chihara et al. This content is distributed under the terms of the Creative Commons Attribution 4.0 International license.

### Sequence and structural motifs from binding sites on mRNAs.

**FIG 2:**
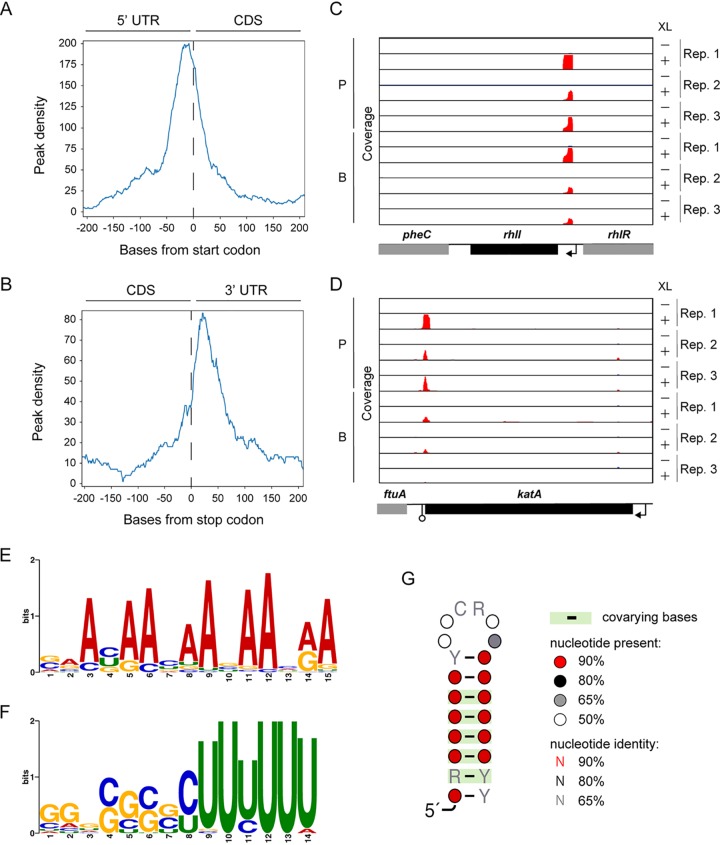
Pseudomonas aeruginosa Hfq binds to AAN triplet repeats on 5′ UTRs and rho-independent terminators. (A and B) Meta-gene analysis along mRNAs with start (A) and stop (B) codons as the reference points. (C and D) Read coverage at the *rhlI* (C) and *katA* (D) loci as representatives of Hfq peaks at the 5′ UTR and 3′ UTR, respectively. TSS (black arrows) and terminator (open circle) annotations were derived from references Gill et al. (39) and Kingsford et al. (40) Rep, replication; XL, cross-linking; P, planktonic; B, biofilm. (E and F) MEME sequence motif analysis for 665 and 617 peaks from the 5′ UTR (E) and 3′ UTR (F) shows the AAN triplet repeats and GC-rich sequences followed by U-rich tails, respectively. (G) CMfinder structural motif analysis of 991 peaks. The highest-scoring motif is shown.

Although P. aeruginosa Hfq is a functional homologue of E. coli Hfq, only the N terminus is highly conserved (22). Initially, to investigate whether P. aeruginosa Hfq, whose CTD is truncated compared to that of E. coli Hfq, also interacts with mRNAs in similar regions to those described for enterobacteria (31, 32), the peak density of Hfq peaks along all detected mRNAs was determined via meta-gene analysis using start or stop codons as reference points. Strong peak densities were observed around both start and stop codons, showing that P. aeruginosa Hfq preferentially binds the 5′ UTRs and 3′ UTRs (Fig. 2A and B). As examples, Hfq binds to the 5′ UTR of *rhlI*, which is translationally upregulated in late exponential phase (24), or the 3′ UTR of *katA*, which putatively functions as a sponge for PrrF1 sRNA (37) (Fig. 2C and D).

Next, 665 and 617 peaks derived from the 5′ UTR and 3′ UTR, respectively, in both planktonic and biofilm data sets were used for MEME motif analysis. Top-ranked motifs from MEME sequence analysis were predicted as five repeats of an AAN triplet at the 5′ UTRs and GC-rich sequences, followed by U-rich tails at the 3′ UTRs (Fig. 2E and F), reminiscent of a Rho-independent terminator. In addition, the top-ranked structural motif from CMfinder was predicted as a highly conserved stem-loop structure (Fig. 2G). We also analyzed sequence and structural motifs in total peaks from planktonic and biofilm conditions separately. These condition-specific analyses showed sequence and structural motifs similar to those of the combined result (Fig. S5).

10.1128/mSystems.00590-19.5 FIG S5 Sequence and structural motif analyses of peaks from individual planktonic and biofilm conditions. In total, 733 and 258 peaks from planktonic and biofilm conditions, respectively, were separately subjected to MEME motif analysis (A) and CMfinder structure motif analysis (B). Adenine- and uracil-rich consecutive sequences were detected as top-ranked sequence motifs. A single stem-loop structure with covarying bases in the stem was detected as a top-ranked structural motif. These consensus motifs were similar under the two conditions. Download FIG S5, TIF file, 0.9 MB. Copyright © 2019 Chihara et al. 2019 Chihara et al. This content is distributed under the terms of the Creative Commons Attribution 4.0 International license.

### Sequence and structural motifs from binding sites on sRNAs.

**FIG 3:**
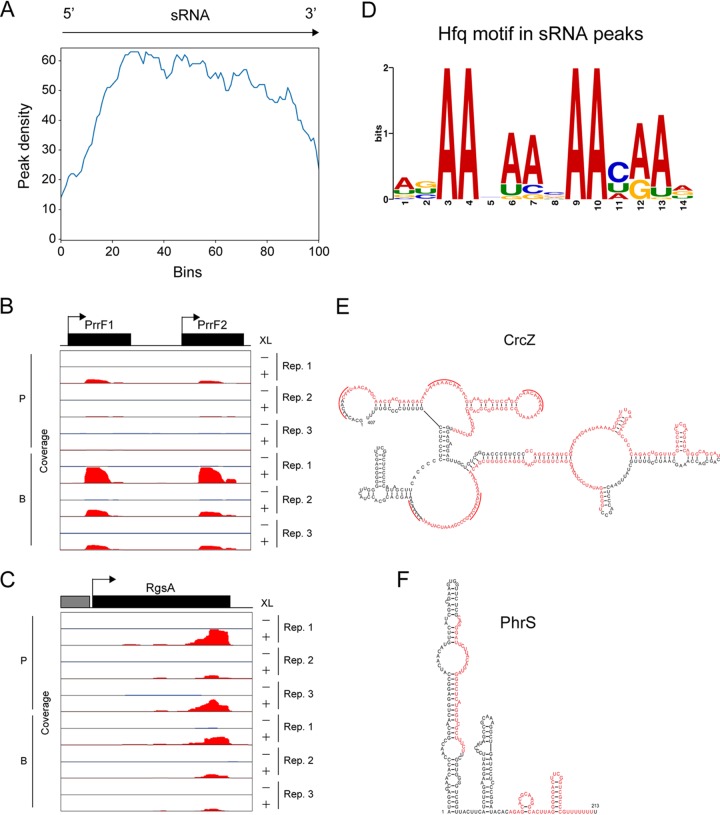
Pseudomonas aeruginosa Hfq binds to the AAN triplet repeats and rho-independent terminators in sRNAs. (A) Meta-gene analysis along sRNAs. Length normalization was achieved through proportional binning in accordance with the different lengths of the sRNA sequences. (B and C) Read coverage at the sRNA PrrF1 (B) and RgsA (C) loci, as indicated, as representatives of Hfq peaks at the central region and the 3′ terminus of sRNAs, respectively. Rep, replication; XL, cross-linking; P, planktonic; B, biofilm. (D) MEME sequence motif analysis for peaks from sRNAs shows the AAN triplet repeats. (E and F) Secondary structures of representative sRNAs CrcZ (E) and PhrS (F). Red letters indicate Hfq cross-linking sites under the planktonic condition. In CrcZ structures, the red arcs indicate previously shown CA repeat motifs (25). Secondary structures were predicted using mfold (69).

Meta-gene analysis for all detected sRNAs indicated that Hfq may preferentially interact with the central region of sRNAs (Fig. 3A). For instance, Hfq peaks in PrrF1/2 sRNAs were identified toward the central region of the transcripts (Fig. 3B), in contrast with *Salmonella* Hfq CLIP-seq conducted previously, wherein Hfq peaks were skewed toward sRNA 3′ ends, particularly toward Rho-independent terminators (32). Nonetheless, Hfq peaks were observed at 3′ ends for some sRNAs in our CLIP-seq analysis. For example, in the region encoding the sRNA RgsA, which posttranscriptionally regulates mRNAs encoding Fis and AcpP (43), a strong peak was detected at the 3′ end (Fig. 3C).

Since Hfq preferentially interacts with GC-rich sequences followed by U-rich tails in 3′ UTRs (Fig. 2F), we speculated that Hfq may interact with the same motif in sRNAs. However, the top-ranked sequence motif in sRNAs was predicted as four repeats of the AAN triplet (Fig. 3D), rather resembling the one detected in 5′ UTR peaks (Fig. 2E). Previously, Sonnleitner et al. reported that the sRNA CrcZ contains CA repeats, which are a hallmark of Crc regulation (25); moreover, CrcZ binds to Hfq and conditionally sequesters it to reduce Hfq-mediated repression of catabolic gene expression (26). Therefore, we hypothesized that CrcZ may also associate with Hfq via CA repeats within the AAN triplet. Three Hfq peaks in CrcZ were detected under the planktonic condition, two of which had AAN triplets in accordance with CA repeat regions (Fig. 3E). In contrast, the general consensus motif for the interaction between Hfq and sRNAs in enterobacteria, namely, the Rho-independent terminator, was also observed at some cross-linking sites. In the region encoding the sRNA PhrS, two Hfq peaks were detected, of which one contained the GC-rich stem-loop followed by a U-rich sequence at the 3′ end (Fig. 3F).

In summary, P. aeruginosa Hfq with a truncated CTD is sufficient to bind to repeats of the triplet AAN, which are present at 5′ UTRs or some sRNAs, and GC-rich stem-loop structures followed by U-rich sequences, which are classical Rho-independent terminators.

### Disentanglement of preferential sRNA affinity to Hfq and expression.

**FIG 4:**
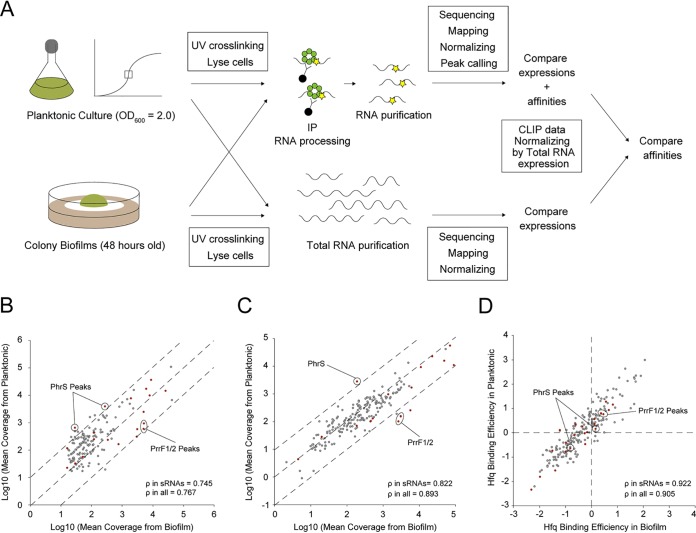
Normalization and comparison of cross-linking immunoprecipitation with high-throughput sequencing of Hfq between planktonic and biofilm conditions. (A) A schematic representation of the comparison of RNA affinities to Hfq between planktonic and biofilm forms. Only overlapping peaks between two phenotypes were considered. Read coverages of cross-linked samples from planktonic conditions were divided by those from biofilms. Concomitantly, read coverages of total RNA from the planktonic condition were divided by those from biofilms. To remove the effect of RNA expression from peak information, fold changes of CLIP-normalized read coverages between planktonic and biofilm conditions were divided by those of total RNA-normalized coverage. (B to D) Mean coverages normalized according to size factors of overlapping peaks detected by CLIP-seq (B) and total RNA-seq (C) between planktonic and biofilm conditions. Dashed lines denote the reference diagonal. (D) Affinity comparison of Hfq normalized peaks on overlapping RNAs between planktonic and biofilm conditions. Dashed lines denote the reference bases. Peaks from sRNAs and others are highlighted in red and gray, respectively. Significantly enriched sRNAs in planktonic (PhrS) or biofilm (PrrF1/2) cultures are indicated with circles. The correlation coefficient ρ was calculated from planktonic versus biofilm.

A number of sRNAs were associated with Hfq under both planktonic and biofilm conditions though the Hfq peak intensities of these sRNAs differed between the two forms (Fig. 3B and C, and Table S1). Since CLIP-seq peaks are a result of both RNA expression and RNA affinity to Hfq under each condition, it is challenging to directly compare CLIP-seq results between different conditions. To disentangle differences in gene expression from those in protein binding, we carried out an *ad hoc* normalization of CLIP-seq using RNA-seq-derived expression measurements (Fig. 4A).

We considered only overlapping peaks between the two conditions. First, read coverage of cross-linked samples under the planktonic condition was divided by coverage from the biofilm condition (“Compare expressions + affinities” denoted in Fig. 4A, and Fig. 4B). Some sRNAs were differentially enriched in the Hfq CLIP-seq between the planktonic and biofilm conditions. For instance, PhrS was more strongly enriched under the planktonic condition (false discovery rate [FDR] of two PhrS peaks, 5.12 × 10^−6^, 2.63 × 10^−4^) (Fig. 4B). Since this sRNA is upregulated from the early stationary phase, depending on the anaerobic sensing factor Anr, and drives PQS/pyocyanin synthesis by upregulating the transcriptional factor MvfR (44), MvfR-regulating genes *pqsABCDE*, *phnAB*, and *phzS* were also transcriptionally upregulated under the planktonic condition (Table S3). In addition, sRNAs P26, CrcZ, ErsA, and NrsZ were also more enriched under the planktonic condition although the levels of enrichment were not statistically significant. In contrast, PrrF1/2 were more strongly enriched under the biofilm condition (FDR of PrrF1/2 peaks, 2.31 × 10^−15^, 6.78 × 10^−17^) (Fig. 4B). P. aeruginosa overcomes iron limitation through pyoverdine/pyochelin production during infection or biofilm development, and PrrF1/2 is in turn associated with these pigments (20, 45, 46). In addition to PrrF1/2, sRNAs Ffs and SsrS were also more enriched under the biofilm condition (FDR for Ffs peak, 2.76 × 10^−12^; FDR for two SsrS peaks, 3.77 × 10^−8^ and 2.59 × 10^−30^).

10.1128/mSystems.00590-19.9 TABLE S3 Differential gene expression based on total RNA-seq. Download Table S3, XLSX file, 1.5 MB. Copyright © 2019 Chihara et al. 2019 Chihara et al. This content is distributed under the terms of the Creative Commons Attribution 4.0 International license.

To control for expression effects, we conducted total RNA-seq from the same planktonic and biofilm cultures used in our CLIP experiments. Considering the normalization biases upon rRNA depletion and the possibility of rRNA binding to Hfq, we did not deplete rRNA. The three biological replicates displayed high correlations within each condition (Fig. S6A and B). DESeq2 comparative analysis of 5,697 transcripts detected via RNA-seq revealed that 478 and 589 RNAs were upregulated under planktonic and biofilm conditions, respectively (FDR, <0.05; log_2_ fold change [FC], >1) (“Compare expressions” denoted in Fig. 4A, and Fig. 4C; see also Fig. S6C and Table S3). Here as well, PhrS and PrrF1/2 were upregulated exclusively in the planktonic and biofilm forms, respectively (FDR of PhrS expression, 6.38 × 10^−34^; FDR of PrrF1/2 expression, 2.83 × 10^−29^, 1.42 × 10^−33^) (Fig. 4C). Overall, CLIP-seq and total RNA-seq results inferred differential sRNA regulation between two phenotypic conditions.

10.1128/mSystems.00590-19.6 FIG S6 Correlation analysis and read coverage distribution based on RNA classes from total RNA sequencing. (A and B) Coefficients of determination *r* and principal components (PC) between biological replicates were calculated from individual gene expression. PC score plots clearly categorized on the basis of planktonic and biofilm conditions. (C) Fold change versus mean reads of total RNA sequencing between planktonic and biofilm conditions. Significantly enriched sRNAs in planktonic (PhrS) or biofilm (PrrF1/2 and PrrH) cultures are indicated with circles. Dashed lines denote the thresholds (log_2_ fold change of >1). Download FIG S6, TIF file, 1.7 MB. Copyright © 2019 Chihara et al. 2019 Chihara et al. This content is distributed under the terms of the Creative Commons Attribution 4.0 International license.

To eliminate the effect of differential RNA expression from Hfq affinity, the fold change in CLIP read coverages between planktonic and biofilm conditions were divided by those of total RNA coverage (Fig. 4A, right, and Fig. 4D). We did not consider non-cross-linked samples because the subjected RNAs were not suggested to be false positives from calculation of both the log_2_ FC(cross-linked sample/non-cross-linked sample) [FC(XL+/XL−)] and the FDR (47, 48). Figure 4D shows that differential sRNA association with Hfq was no longer seen after the normalization based on total RNA-seq, implying that the Hfq-sRNA affinity remains constant across conditions. In fact, while the correlation coefficient ρ of sRNAs from CLIP-seq and total RNA-seq between conditions is relatively low (ρ in sRNAs, 0.745 and 0.822) (Fig. 4B and C), the correlation in Hfq-binding efficiency is high (ρ in sRNAs, 0.922) (Fig. 4D). These results suggest that the primary driver of differential sRNA association with Hfq is expression rather than differences in relative affinities due to changing RNA pools. This lends support to a model of RNA concentration-driven cycling of Hfq, known as the associative/active cycling model. In this model, free RNA transiently binds to the Hfq-RNA complex, with frequent RNA exchange preventing Hfq availability from becoming a limiting factor in regulation under most conditions (5).

## DISCUSSION

Gram-negative P. aeruginosa is ubiquitously distributed in diverse environments. Upon biofilm formation in the human body, this bacterium causes chronic biofilm-related infections in burn wounds, in *in vivo*-dwelling devices, and in the lungs of immunocompromised patients with cystic fibrosis (12). Although posttranscriptional regulatory networks that enable survival in dynamic environments are known to be driven by sRNAs and associated RNA chaperones such as Hfq, RsmA, Crc, and the newly identified ProQ (2), limited information is available regarding the global role of RNA chaperones in the P. aeruginosa transcriptome, especially under different environmental conditions. CLIP-seq analysis, which helps decipher RNA-RBP interactions with RNA targets, is well suited to explore regulatory functions of RBPs depending on a cell’s physiological state (31–33).

In this study, we performed comparative CLIP-seq analysis between early stationary planktonic cultures and colony biofilms, thus elucidating condition-specific RNA regulation by Hfq. Consequently, 991 peaks with significant enrichment in cross-linked samples were identified throughout the P. aeruginosa PAO1 genome although fewer peaks were observed under the biofilm condition than under the planktonic condition (Fig. 1A to D). RNA abundance decreases under biofilm conditions, with a concomitant reduction in bacterial growth (49). Nevertheless, biofilm-specific Hfq-RNA interactions were observed even at a low peak number (Fig. 1C to E). Previously, Pusic et al. reported that CrcZ is the most abundant sRNA bound to Hfq in P. aeruginosa PA14 anoxic biofilm and indirectly impacts biofilm formation (50). On the other hand, our CLIP-seq result shows that CrcZ binds to Hfq under the planktonic condition rather than the biofilm condition (see Table S1 in the supplemental material). The difference in Hfq-binding sRNAs between two studies may come from the difference in the biofilm conditions. Pusic et al. formed the anoxic biofilm in polypropylene tubes supplemented with synthetic cystic fibrosis sputum medium, and this condition may stimulate CrcZ expression through the anaerobic sensing factor Anr. In contrast, in the current study, colony biofilms were formed on LB agar aerobically, and this condition may repress CrcZ expression due to the availability of a preferred carbon source. The present effort, as a result, illuminated multifaceted roles of Hfq underlying the dynamic regulations of biological processes between different physiological conditions, including different sorts of biofilms.

Since P. aeruginosa Hfq can successfully complement its E. coli ortholog, it is a functional homologue of E. coli Hfq (51). The association of P. aeruginosa Hfq with RNA was investigated on the basis of sequence/structure motifs and the general distribution of detected peaks (Fig. 2). Meta-gene analysis of peak distribution among mRNAs mirrored previously described Hfq-binding preferences in *Salmonella* (32), indicating that Hfq peaks are highly enriched at both the 5′ UTR and 3′ UTR (Fig. 2A to D). In addition, sequence and structural motif analyses revealed that five AAN triplet sequence repeats at the 5′ UTRs and the stem-loop structure, followed by the U-rich tail at the 3′ UTRs are preferential binding sites for Hfq (Fig. 2E to G). The sequence motif from the 3′ UTRs corresponds to Rho-independent terminators previously identified by *Salmonella* Hfq CLIP-seq (32). On the other hand, while the results derived from the 5′ UTRs are congruent with previous reports regarding the distal surface of E. coli Hfq-binding A-rich sequences *in vitro* (52), this is the first evidence of AAN triplet sequence repeats in mRNAs binding Hfq *in vivo*.

P. aeruginosa Hfq lacks a long CTD, unlike E. coli and *Salmonella* Hfq. The mechanism of action of the CTD in Hfq remains controversial; however, the CTD of E. coli Hfq is required for the release of annealed mRNA-sRNA double-stranded conjugates from the rim surface (53, 54). Since a truncated CTD wraps over the rim of P. aeruginosa Hfq and since there is no association with the proximal and distal surfaces (53, 54), the present results can be interpreted that AAN triplets at the 5′ UTRs and GC-rich stem-loop followed by U-rich tails at the 3′ UTRs are common binding sites across bacterial orders, at least between *Enterobacteriales* and *Pseudomonadales*, regardless of the length of the CTDs. Although lack of a CTD strengthens the RNA association on rim surface, a UA-rich consensus motif for the rim surface was indiscernible from our CLIP-seq, suggesting that a truncated CTD may be enough to release mRNA-sRNA conjugates (55).

Interestingly, the peak distributions among sRNAs were relatively broad (Fig. 3A), and motif analysis revealed that four AAN triplet repeats in sRNAs are preferential binding sites for Hfq (Fig. 3D). In enterobacteria, sRNAs have been divided into so-called class I and class II sRNAs based on their sequence conservation and mode of interaction with Hfq, among which class II sRNAs contain AAN motifs in the stretched sequence, as well as U-rich tails at the 3′ ends, and are more stable than class I sRNAs (6). Although the majority of E. coli sRNAs are class I sRNAs, the prediction of AAN triplet repeats implies a higher relative abundance of the class II sRNAs in the P. aeruginosa transcriptome. Interestingly, the CTD of E. coli Hfq has been suggested to offer a binding advantage specifically to class II sRNAs (53). Why does P. aeruginosa, which has a short Hfq CTD compared with that of E. coli, seem to preferentially express class II sRNAs? One answer may come from recent structural analysis of the association of Crc as the fourth partner with the sRNA-Hfq-mRNA ternary regulatory complex. Crc itself does not interact with sRNAs through Rho-independent terminators; however, it does enhance the association between Hfq and A-rich target sequences via an Hfq (distal surface)-RNA-Crc sandwich conformation (27, 56), perhaps thus potentially selecting for the AAN triplet motif. It may be interesting to consider the possibility that sRNAs with AAN motifs tend to regulate mRNAs with U-rich motifs. However, Schu et al. reported that a chimeric sRNA ChiX-RyhB in which the 5′ end of ChiX with an AAN motif was fused to RyhB with a U-rich motif reduces the negative regulation of RyhB target mRNA *sodB* with AAN motif and that another chimeric sRNA, ChiXΔAAN-RyhB-ChiX, which lacks an AAN motif in the ChiX region, restores the negative regulation to *sodB* in E. coli (6). This suggests that sRNA cannot regulate its target when each binding site to Hfq competes. Therefore, a class II sRNA with both an AAN motif and U-rich motif may not regulate a target with a U-rich motif as long as the binding of U-rich sequences in both the sRNA and the target RNA to proximal surface of Hfq competes.

CLIP-seq converts read counts obtained from Illumina sequencing to peaks. Because obtained peaks are products of both RNA expression and RNA affinity, it is difficult to directly compare CLIP-seq results between different phenotypes or conditions. To circumvent this issue, we carried out an *ad hoc* normalization of CLIP-seq results based on RNA expression (Fig. 4). Disentanglement from background expression suggested that the association of certain sRNAs to Hfq is primarily altered based on their differential expression (Fig. 4A to C), a fact reminiscent of the associative/active cycling model of Hfq-RNA interactions (5). Generally, several mechanisms may underlie changes in RNA association with Hfq, depending on physiological states. First, since *hfq* expression levels did not significantly differ between planktonic and biofilm conditions (Table S3), competition among sRNAs for invariable binding sites on Hfq may have occurred. Second, sRNA association can be altered depending on the variations in target RNA expression in each environment. This target-centric view is reminiscent of condition-specific base paring between miRNA and microRNA response elements in higher eukaryotes (57). Finally, the turnover rate of complex formation potentially affects the RNA-Hfq association. Interactions between RBP and RNA were previously reported to last 2 to 3 min *in vivo*; however, they were robust *in vitro*, with a half-life of >100 min, owing to turnover of complex formation depending on the levels of free RNAs (5). In other words, while RNA with a low abundance can temporarily bind RBP, it is replaced with highly abundant RNA. Additionally, another RBP, Crc, enhances Hfq association with AAN triplet repeats in P. aeruginosa (56). Therefore, the Hfq interactome may be strengthened with RNAs, wherein the interaction with AAN triplets is rigid. While the current study cannot unravel which mechanisms in RNA association with Hfq are preferential *in vivo*, our combinatorial approach shows that the affinity of an individual sRNA does not change and that the association with Hfq is just dependent on expression differences. Although one of the causes which make the understanding of the mechanism in RNA association with Hfq *in vivo* difficult is lacking in complete list of sRNA-Hfq-mRNA interactions, recent progress in the study about sRNA-mRNA interactome is encouraging using *in vivo* preferential ligation of RNAs (36). We could find a clue of a molecular mechanism of action of Hfq by comparing Hfq-binding efficiency among the different conditions provided in the present study with comprehensive sRNA interactome studies.

Finally, we have identified more than 100 putative sRNAs bound to Hfq in either or both of the planktonic and biofilm forms with manual curation of sRNAs from size selection sRNA-seq conducted previously (16, 17) (Table S1). The evidence that these sRNAs bind to Hfq suggests that they may be functional, not just RNA degradation products. Further experiments will be needed to understand the mechanism of action of the novel Hfq-binding sRNAs in P. aeruginosa. Altogether, the present combinatorial comparative CLIP-seq and total RNA-seq approach uncovered condition-dependent association of sRNAs with Hfq *in vivo* and dramatically expanded the potential direct regulatory targets of Hfq in the P. aeruginosa transcriptome.

## MATERIALS AND METHODS

### Strains, plasmids, and growth conditions.

Strains, plasmids and oligonucleotides used here are enlisted in Table S4 in the supplemental material. All experiments were performed using P. aeruginosa PAO1 or its derived strains. Each strain was cultured at 37°C in Luria-Bertani (LB) medium.

10.1128/mSystems.00590-19.10 TABLE S4 The list of strains, plasmids, and oligonucleotides. Download Table S4, DOCX file, 0.03 MB. Copyright © 2019 Chihara et al. 2019 Chihara et al. This content is distributed under the terms of the Creative Commons Attribution 4.0 International license.

### Construction of FLAG-tagged Hfq-harboring and *hfq* deletion strains.

PAO1 *hfq*::3×FLAG and Δ*hfq* strains were constructed through conjugative transfer of appropriate plasmids and homologous recombination between the chromosome and plasmid DNA as previously described (58). pG19*hfq*::3×FLAG was constructed by cloning PCR products at HindIII/BamHI sites 1 kb upstream of the *hfq* stop codon in the PAO1 chromosome, three copies of a FLAG tag, and 1 kb downstream of the *hfq* stop codon in the PAO1 chromosome into the pG19II backbone. pG19Δ*hfq* was constructed by cloning PCR products at HindIII/XbaI sites 500 bp upstream of the *hfq* start codon and 500 bp downstream of the *hfq* stop codon in the PAO1 chromosome into the pG19II backbone.

### UV cross-linking, immunoprecipitation, and RNA purification.

For the planktonic condition, 200-ml bacterial cultures of three replicates were maintained up to an OD_600_ of 2.0 in LB medium. For the biofilm condition, an overnight culture was diluted 100-fold, and 10 μl of the aliquot was seeded on a cellulose membrane (HATF02500; Millipore) placed on the LB agar. Before the cellulose membrane was placed on LB agar, it was UV irradiated on both sides for 10 min each for surface sterilization. Colony biofilms were incubated statically for 48 h at 37°C. Membranes with colony biofilms were aseptically transferred to fresh LB agar every 24 h. Because the OD_600_ could not be determined for colony biofilms, we measured the weight of pellets from both planktonic (OD_600_ of 2.0) and 48-h-incubated colony biofilms and determined the number of necessary colony biofilms for CLIP experiments. After 48 h of incubation, 36 colony biofilms from the same biological replicate were scraped with a sterilized loop into 200 ml of LB medium. UV cross-linking and immunoprecipitation were performed as previously described (32, 34). Briefly, half of the culture from each condition was irradiated at 800 mJ of UV light at 254 nm. After UV cross-linking, the sample, as well as non-cross-linked control samples, was centrifuged for 20 min at 4,700 rpm at 4°C. Cell pellets were lysed in a Retch mill at 30 Hz for 10 min with 1 ml of 0.1-mm glass beads and 800 μl of NP-T buffer (50 mM NaH_2_PO_4_, 300 mM NaCl, 0.05% Tween 20, pH 8.0). NP-T buffer supplemented with 8 M urea was added to each supernatant at an equal volume and incubated for 5 min at 65°C with agitation at 900 rpm. Anti-FLAG magnetic beads were washed three times with 500 μl of NP-T buffer, resuspended in 125 μl of NP-T buffer, and treated with a 120-μl suspension of urea-treated samples. After 1 h of incubation at 4°C, samples were washed twice with 500 μl of high-salt buffer (50 mM NaH_2_PO_4_, 1 M NaCl, 0.05% Tween 20, pH 8.0), followed by two washes with 500 μl of NP-T buffer. Beads were resuspended in Benzonase mix (500 units of Benzonase nuclease [E1014; Sigma-Aldrich] in NP-T buffer with 1 mM MgCl_2_) and incubated for 10 min at 37°C with agitation at 900 rpm. After one wash with high-salt buffer and two washes with calf intestinal alkaline phosphatase (CIP) buffer (100 mM NaCl, 50 mM Tris-HCl, pH 7.4, 10 mM MgCl_2_), beads were resuspended in 200 μl of CIP mix (20 units of CIP [M0290; NEB] in CIP buffer) and incubated for 30 min at 37°C with agitation at 800 rpm. After one wash with high-salt buffer and two washes with polynucleotide kinase (PNK) buffer (50 mM Tris-HCl, pH 7.4, 10 mM MgCl_2_, 0.1 mM spermidine), beads were resuspended in 100 μl of PNK mix (10 units of T4 PNK [EK0032; ThermoFisher Scientific], 10 μCi of [γ-^32^P]ATP in PNK buffer) and incubated for 30 min at 37°C, followed by addition of 10 μl of 1 mM nonradioactive ATP and incubation for 5 min at 37°C. After two washes with NP-T buffer, beads were resuspended in 30 μl of protein loading buffer and incubated for 5 min at 95°C. Beads were magnetized and supernatants were transferred to fresh tubes.

Five-microliter aliquots and the rest of the supernatants were loaded and separated via SDS-polyacrylamide gel electrophoresis (12% resolving gel) for 1.5 h at 340 mA, followed by Western blot analysis and RNA extraction. After electrophoresis, proteins were electrotransferred onto a polyvinylidene difluoride membrane, which was blocked in 1× TBS-T buffer (20 mM Tris, 150 mM NaCl, 0.1% Tween 20, pH 7.6) with 10% skim milk for 45 min. Thereafter, the membrane was probed overnight at 4°C with monoclonal anti-FLAG (31430, 1:1,000; ThermoFisher Scientific) antibody diluted in 1× TBS-T buffer containing 3% bovine serum albumin, washed three time for 5 min each in 1× TBS-T buffer, probed for 1 h with anti-mouse horseradish peroxidase (HRP) antibody (F1804, 1:10,000; Sigma-Aldrich ) diluted in 1× TBS-T buffer containing 3% bovine serum albumin, and washed three times for 5 min each time in 1× TBS-T buffer. Chemiluminescent signals were detected using Image Quant LAS 4000 (GE Healthcare).

After RNA electrophoresis, the RNA was transferred from the gel to Protran membrane (10600016; GE Healthcare). The membrane was placed in a cassette with a phosphor screen and exposed overnight. The autoradiogram was printed on clear paper and aligned with the membrane, and bands were cut out. Each membrane piece was cut into smaller pieces and placed in 2 ml of LoBind tubes with 400 μl of proteinase K (PK) solution (1.3 mg/ml PK [EO0491; ThermoFisher Scientific], 10 units of RNase inhibitor [10777019; Invitrogen] in 2× PK buffer [100 mM Tris-HCl, pH 7.9, 10 mM EDTA, 1% SDS]), followed by incubation for 1 h at 37°C with agitation at 1,000 rpm and then by incubation for 1 h at 37°C with agitation at 1,000 rpm with 100 μl of PK buffer with 9 M urea. Thereafter, 450 μl of supernatants from proteinase K-treated membranes was mixed with an equal volume of phenol-chloroform-isoamyl alcohol in phase-lock gel tubes and incubated for 5 min at 30°C with agitation at 1,000 rpm. Each mixture was centrifuged for 12 min at 13,000 rpm at 4°C, and 400 μl of the aqueous phase was precipitated with 3 volumes of ice-cold ethanol, 1/30 volume of 3 M sodium acetate (NaOAc; pH 5.2), and 1 μl of GlycoBlue (AM9515; Invitrogen) for 2 h at –20°C. The precipitated pellet was washed with 80% ethanol, briefly dried for 5 min at 20°C, and resuspended in 11 μl of sterilized water.

### Total RNA purification.

Two-hundred microliters of stop solution (95% [vol/vol] ethanol and 5% [vol/vol] water-saturated phenol, pH > 7.0) was added into 1-ml aliquots of each cross-linked culture and immediately incubated at –80°C. The frozen culture was defrosted on ice and centrifuged for 20 min at 4,500 rpm at 4°C. Total RNA was extracted via the hot phenol extraction method. Briefly, the culture containing the stop solution in the tube was resuspended in a mixture containing 600 μl of Tris-EDTA (TE) buffer, 0.5 mg/ml lysozyme (pH 8.0), and 60 μl of 10% (wt/vol) SDS. The tube was placed in a water bath for 5 min at 65°C, followed by addition of 66 μl of NaOAc (pH 5.2). Thereafter, 750 μl of phenol was added into the tube and mixed via tube inversion every 30 s during incubation for 5 min at 65°C. The tube was centrifuged for 15 min at 13,000 rpm at 4°C. The aqueous phase was transferred to a phase-lock gel tube, and 750 μl of chloroform was added. After samples were mixed via tube inversion, the mixture was centrifuged for 12 min at 13,000 rpm at 4°C. The aqueous phase was precipitated with 2 volumes of ice-cold ethanol and 1/30 volume of 3 M NaOAc (pH 6.5) for 2 h at –20°C. The precipitated pellet was washed with ice-cold 75% ethanol, briefly dried for 5 min at 20°C, and resuspended in 50 μl of sterilized water. Thereafter, 10 and 40 μg (vol/wt) of RNA were extracted from biofilm and planktonic cultures, respectively, in 39.5 μl of sterilized water. A total of 10.5 μl of DNase solution (5 units of DNase I and 5 units of RNase inhibitor in DNase buffer) was added to each sample and incubated for 30 min at 37°C, and 100 μl of sterilized water was added. Thereafter, 150 μl of the total mixture was transferred to a phase-lock gel tube with 150 μl of phenol-chloroform-isoamyl alcohol. After the samples was mixed via tube inversion, the mixture was centrifuged for 15 min at 13,000 rpm at 4°C. The aqueous phase was precipitated with 2.5 volumes of ice-cold ethanol and 1/30 volume of 3 M NaOAc (pH 6.5) for 2 h at –20°C. The pellet was washed with ice-cold 75% ethanol, briefly dried for 5 min at 20°C, and resuspended in 40 μl of sterilized water. RNA quantity and quality were determined via a NanoDrop spectrophotometer and electrophoresis on a 1% (wt/vol) agarose gel, respectively.

### cDNA library preparation and sequencing.

A cDNA library of the CLIP samples was prepared using an NEBNext Multiplex Small RNA Library Prep Set for Illumina (E7300; NEB) in accordance with the manufacturers’ instructions with certain modifications. First, a 3′ SR adapter and 5′ SR adapter (Illumina) were diluted 10-fold with nuclease-free water before use. Second, each reagent was prepared at half the volume instructed by the manufacturer. Third, 2.5 μl of each purified RNA was used for cDNA library preparation. PCR amplification was performed for cDNA under the following conditions: 18, 20, and 22 cycles for initial amplification and 23 cycles during the final amplification. cDNA libraries were quantified using a Qubit double-stranded DNA (dsDNA) assay (Q32854; Invitrogen) and Agilent 2100 Bioanalyzer dsDNA assay (Agilent). High-throughput sequencing was performed at Core Unit Systems Medicine, University of Würzburg, Würzburg, Germany. Twelve cDNA libraries from CLIP were pooled on an Illumina NextSeq 500 mid-output flow cell and sequenced in paired-end mode (2 × 75 cycles). For total RNA sequencing, high-throughput sequencing was performed at Vertis Biotechnologie AG, Freising, Germany. cDNA libraries were prepared from total RNA and pooled on an Illumina NextSeq 500 mid-output flow cell and sequenced in single-end mode (1 × 75 cycles).

### Sequence processing and mapping.

For CLIP-seq, to ensure high sequence quality, read 1 (R1) and read 2 (R2) files containing the Illumina paired-end reads in FASTQ format were quality and adapter trimmed via Cutadapt (59), version 1.15/1.16, using a cutoff Phred score of 20 in NextSeq mode, and reads without any remaining bases were discarded (command line parameters: --nextseq-trim = 20 -m 1 -a AGATCGGAAGAGCACACGTCTGAACTCCAGTCAC -A GATCGTCGGACTGTAGAACTCTGAACGTGTAGATCTCGGTGGTCGCCGTATCATT). To eliminate putative PCR duplicates, paired-end reads were collapsed using FastUniq (60). After trimming, we applied the pipeline READemption (61), version 0.4.5, to align all reads longer than 11 nt to the P. aeruginosa PAO1 chromosome (NCBI accession no. NC_002516.2) reference genome using segemehl (62), version 0.2.0, with an accuracy cutoff of 80%. From the results, only those reads mapping uniquely to one genomic position were considered for all subsequent analyses.

For RNA-seq, to ensure high sequence quality, Illumina reads were quality and adapter trimmed via Cutadapt (59), version 1.15, using a cutoff Phred score of 20 in NextSeq mode, and reads without any remaining bases were discarded (command line parameters: --nextseq-trim = 20 -m 1 -a AGATCGGAAGAGCACACGTCTGAACTCCAGTCAC). After sequences were trimmed, we applied the pipeline READemption (61), version 0.4.5, to align all reads longer than 11 nt to the P. aeruginosa PAO1 (NCBI accession no. NC_002516.2) reference genome using segemehl, version 0.2.0 (62), with an accuracy cutoff of 95%.

### Transcript and UTR annotations.

The transcript and subsequently the UTR annotations were generated for the planktonic and biofilm RNA-seq data with the pipeline ANNOgesic (41), version 0.7.33. Therefore, the annotation reference file for P. aeruginosa PAO1 (NCBI accession no. NC_002516.2), the reported TSS based on differential RNA-seq data (39), and the terminators detected by ANNOgesic (41) via TransTermHP (40) were used. The transcripts were adjusted based on the reference genome annotation positions. The 3′ UTRs were annotated based on the transcripts as well as terminators, and the 5′ UTR annotations were based on the TSS and transcripts. Other parameters were set to default.

For all analyses related to annotated genomic features such as CDSs and tRNAs, gene annotations from PseudoCAP (http://www.pseudomonas.com) were considered.

### Read count normalization and peak calling based on CLIP-seq.

Read counts per position were exploratorily analyzed to isolate the core of positions present in cross-linked and non-cross-linked library pairs, as described previously (34). The areas with low read counts were filtered from both cross-linked and non-cross-linked libraries using a standard deviation of 6 or less as an index. After the difference in read counts between the two libraries was plotted, the size factor was calculated using the DESeq normalization procedure from the high-count positions in both libraries across all replicates (63).

We applied PEAKachu, version 0.1.0 (https://github.com/tbischler/PEAKachu), for the peak calling in a similar way as described previously (34). First, BAM files for the respective pairs of cross-linked and non-cross-linked libraries were used to run in paired-end (-P) and paired-replicate (-r) mode. The maximum fragment size (-M) was set to 50, and annotations generated as GFF format (see above) were used to map overlapping features to the called peaks. Normalization was performed in the ‘manual’ mode using previously determined size factors (see above). Other parameters were set to default. Second, the boundary of initial peaks was set through block definition computed by the blockbuster algorithm (64) based on pooled read alignments from all cross-linked libraries using default parameters. Third, the PEAKachu tool runs DESeq2 (65) to analyze the significance of peak enrichment in the cross-linked libraries relative to levels in the non-cross-linked libraries with parameter values as follows: mad-multiplier (-m), 1.0; fold change (-f), 1.0; and adjusted *P* value (-Q), 0.05. Finally, PEAKachu was used for each replicon and strand to generate normalized coverage plots for the facilitation of data visualization.

### Differential expression analysis based on total RNA-seq.

We applied READemption (61) to assess the overlap of read alignments for each library to the same annotations used in the CLIP-seq analysis on the sense strand. Each read with a minimum overlap of 1 nt was counted with a value based on the number of locations where the read was mapped. If the read overlapped more than one annotation, the respective value was counted once for each overlapping region. The resulting read counts were subjected to differential expression analysis of planktonic versus biofilm samples via DESeq2 (65), version 1.18.1. Fold change shrinkage was applied by setting the parameter ‘betaPrior=TRUE’.

### Analysis of sequence and structural motifs.

The sequences of peaks from planktonic, biofilm, and combined conditions were used to perform MEME sequence motif analysis (66). Minimum and maximum motif widths were set at 6 and 50, respectively, while other parameters were set to default.

Structural motifs of the sequences of peak regions extended by an additional 10 nt upstream and downstream from planktonic, biofilm, and combined conditions were analyzed using CMfinder, version 0.2.1 (67). The minimum length of single stem-loop candidates was set as 20, while other parameters were set to default. Each analyzed motif was visualized using R2R (68).

### Statistical and other analysis.

Descriptive statistical analyses for peak overlapping between two conditions, peak distributions across the P. aeruginosa genome, and peak classification among RNA classes were performed using Microsoft Excel. A peak density plot was constructed using Python 3. Genes identified via CLIP-seq analysis were functionally characterized using the PseudoCAP annotation (http://www.pseudomonas.com). KEGG enrichment analysis was performed from genes identified via CLIP-seq analysis using DAVID (https://david.ncifcrf.gov/summary.jsp). The default parameters and databases were used, and multiple testing adjustments were performed using the Benjamini-Hochberg method.

### Data availability.

Raw sequencing reads in FASTQ format are available in NCBI’s Gene Expression Omnibus (GEO [https://www.ncbi.nlm.nih.gov/geo]) under accession number GSE136112.
