# Supplementary figures and images for: Conditional Hfq Association with Small Noncoding RNAs in Pseudomonas aeruginosa Revealed through Comparative UV Cross-Linking Immunoprecipitation Followed by High-Throughput Sequencing
Source: mSystems. 2019 Dec 3;4(6):e00590-19. doi: 10.1128/mSystems.00590-19 (PMC6890931; doi:10.1128/mSystems.00590-19)

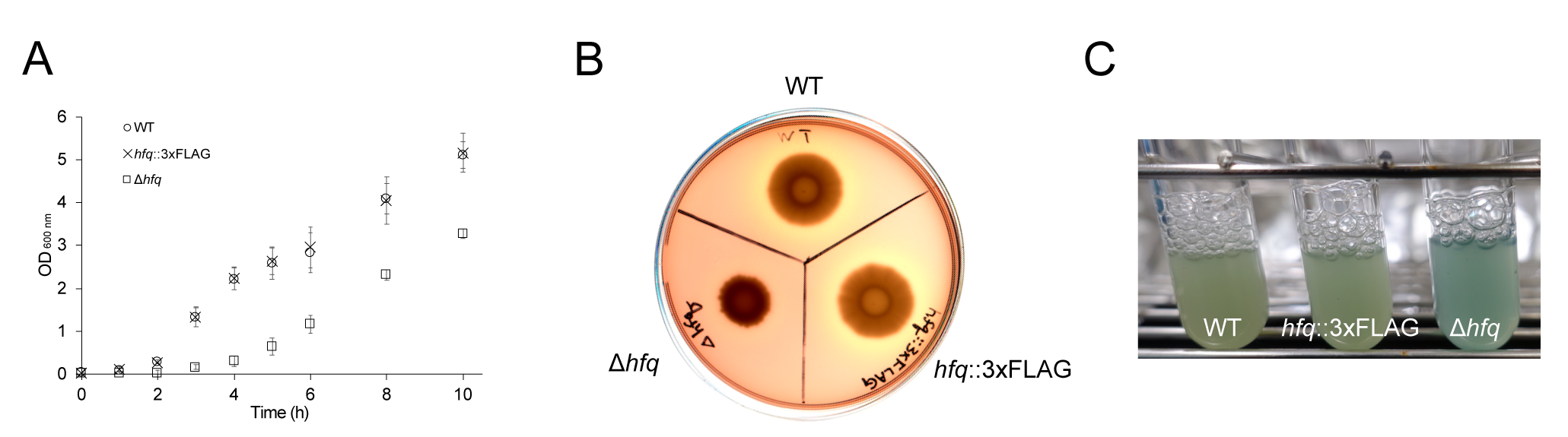

Supplement: FIG S1 [file mSystems.00590-19-sf001.tif]

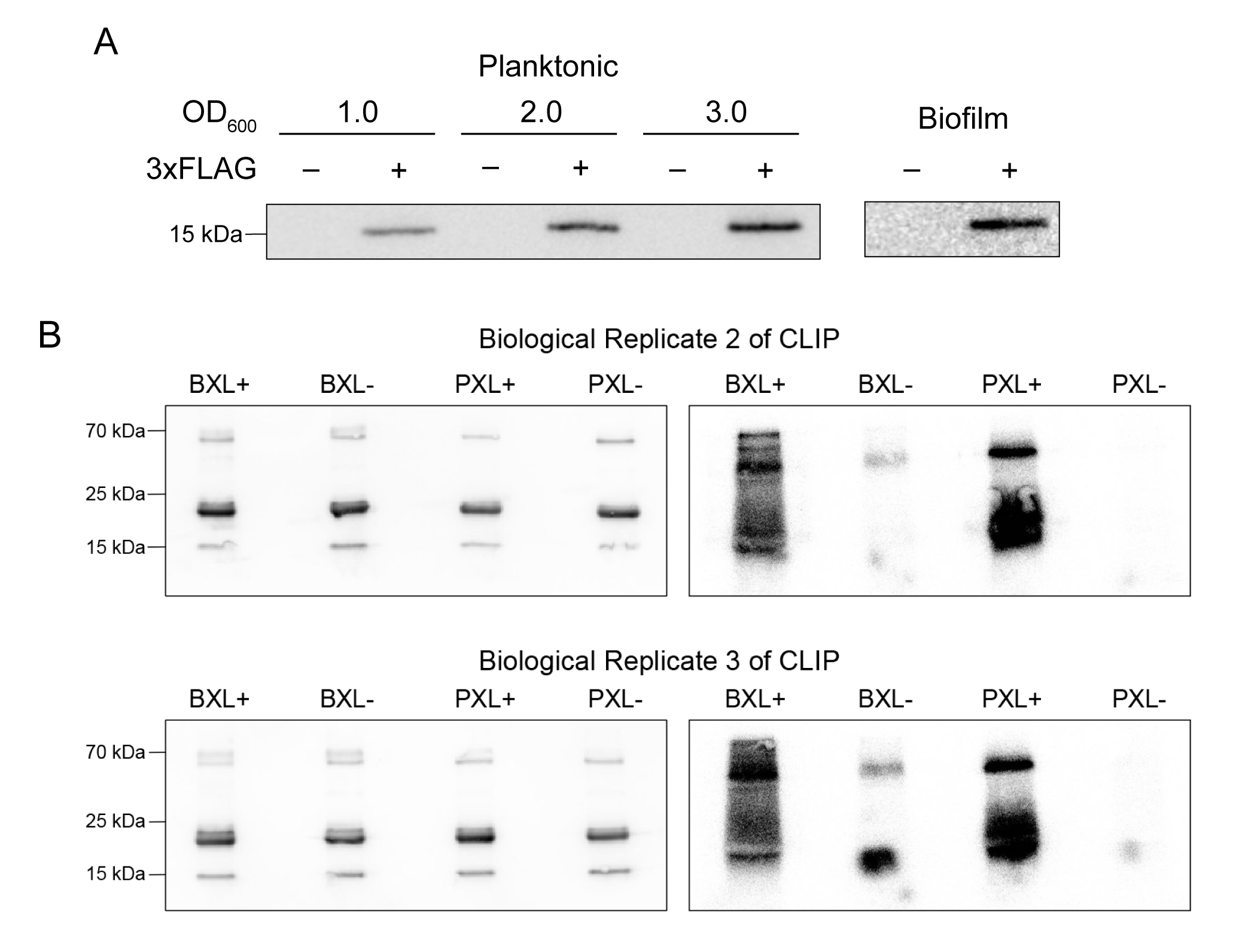

Supplement: FIG S2 [file mSystems.00590-19-sf002.tif]

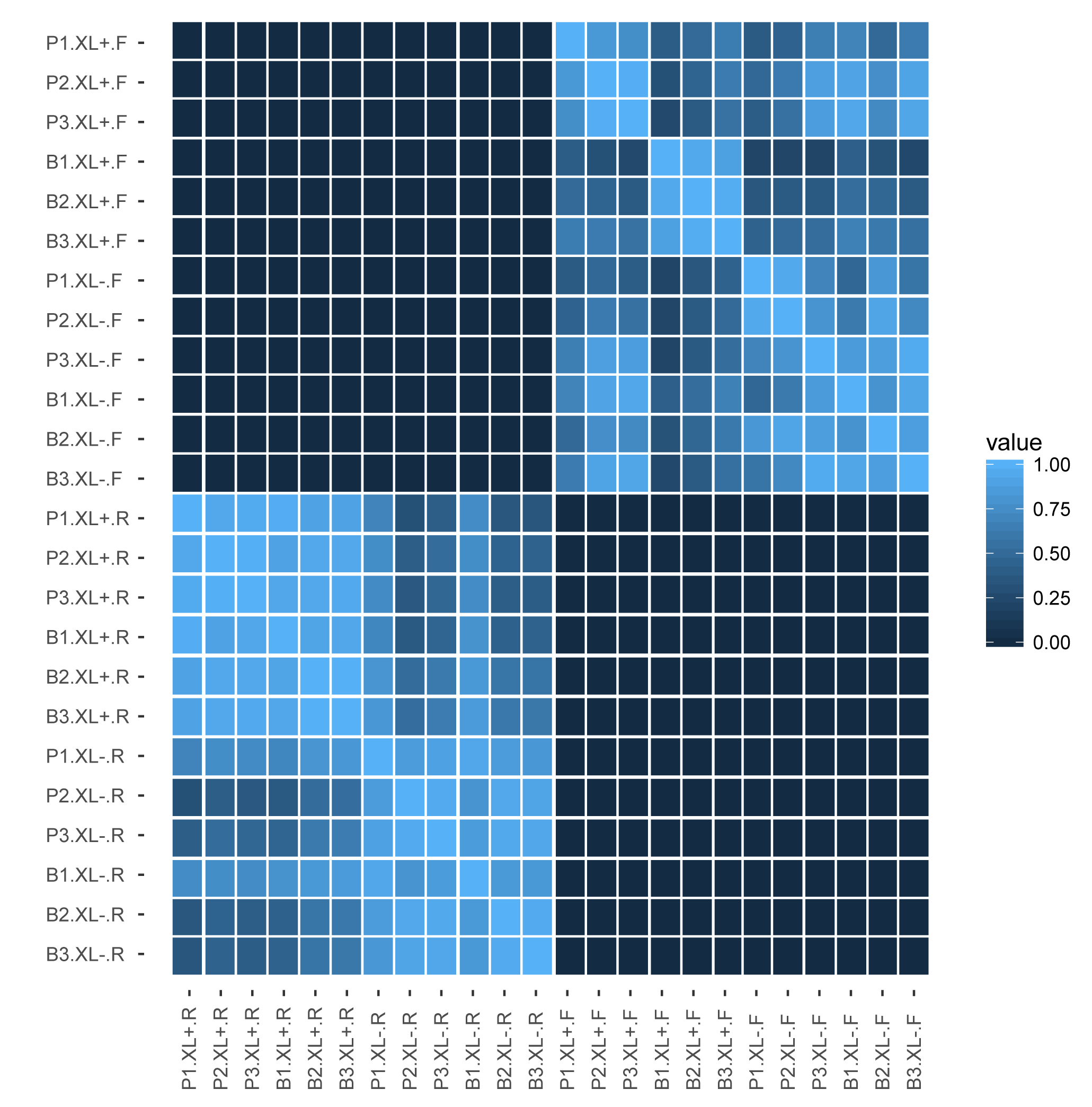

Supplement: FIG S3 [file mSystems.00590-19-sf003.tif]

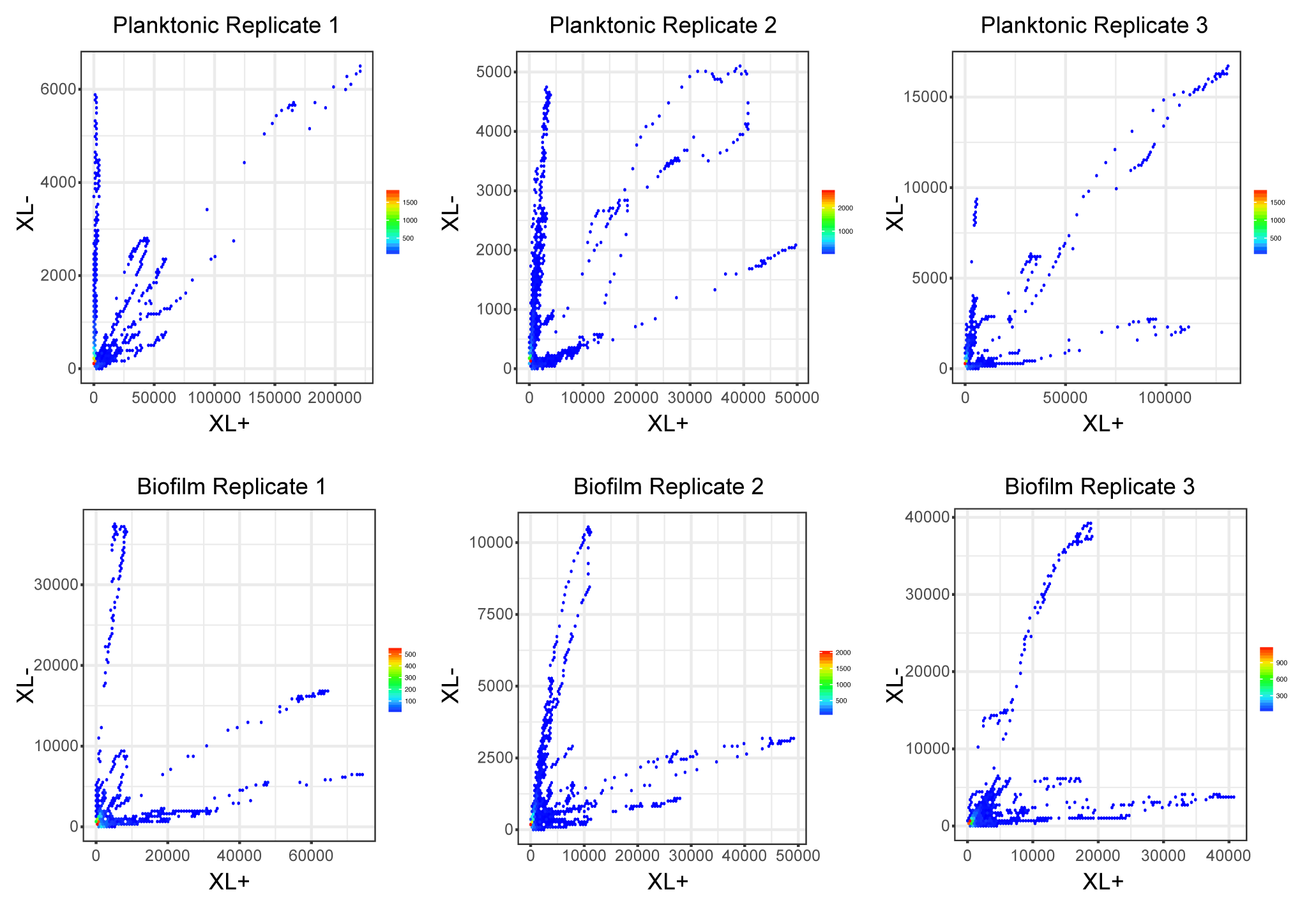

Supplement: FIG S4 [file mSystems.00590-19-sf004.tif]

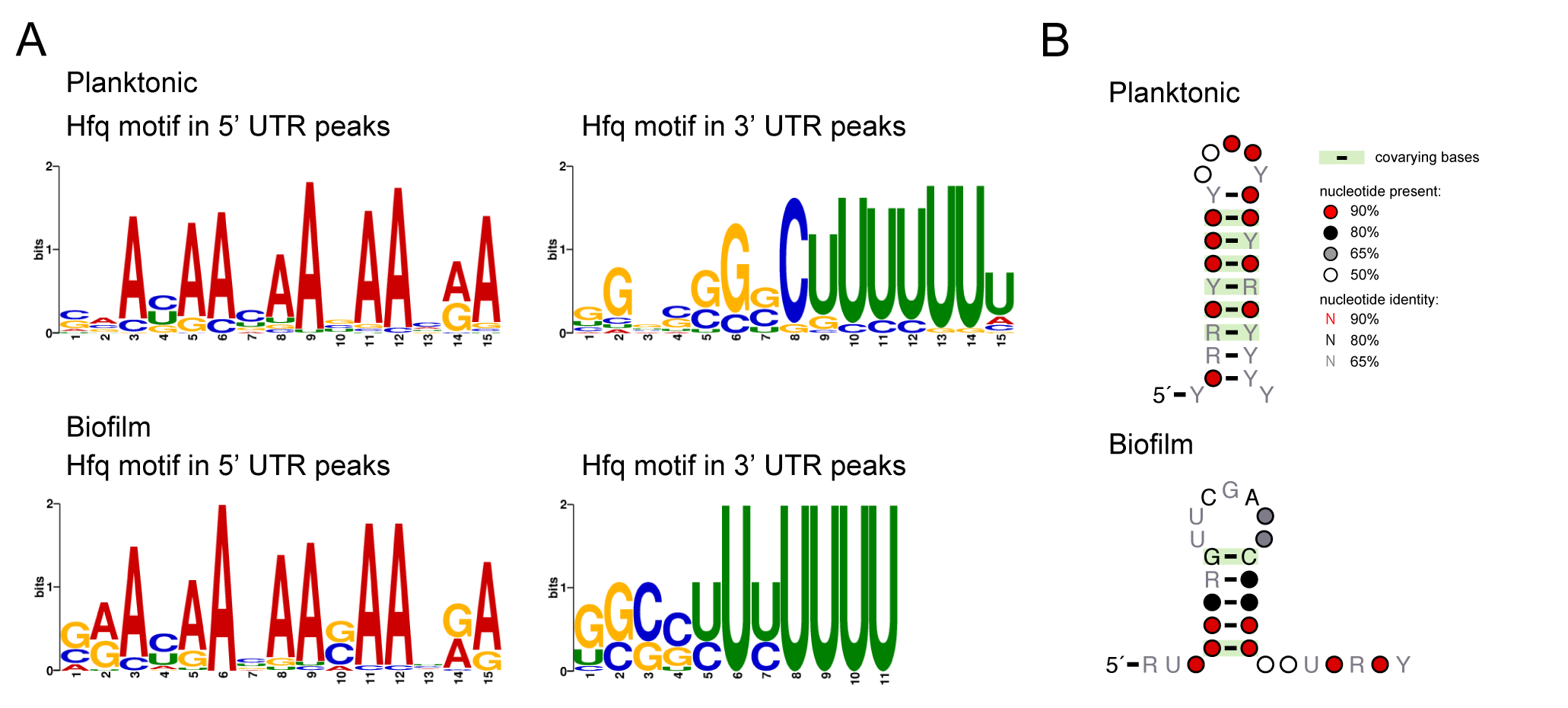

Supplement: FIG S5 [file mSystems.00590-19-sf005.tif]

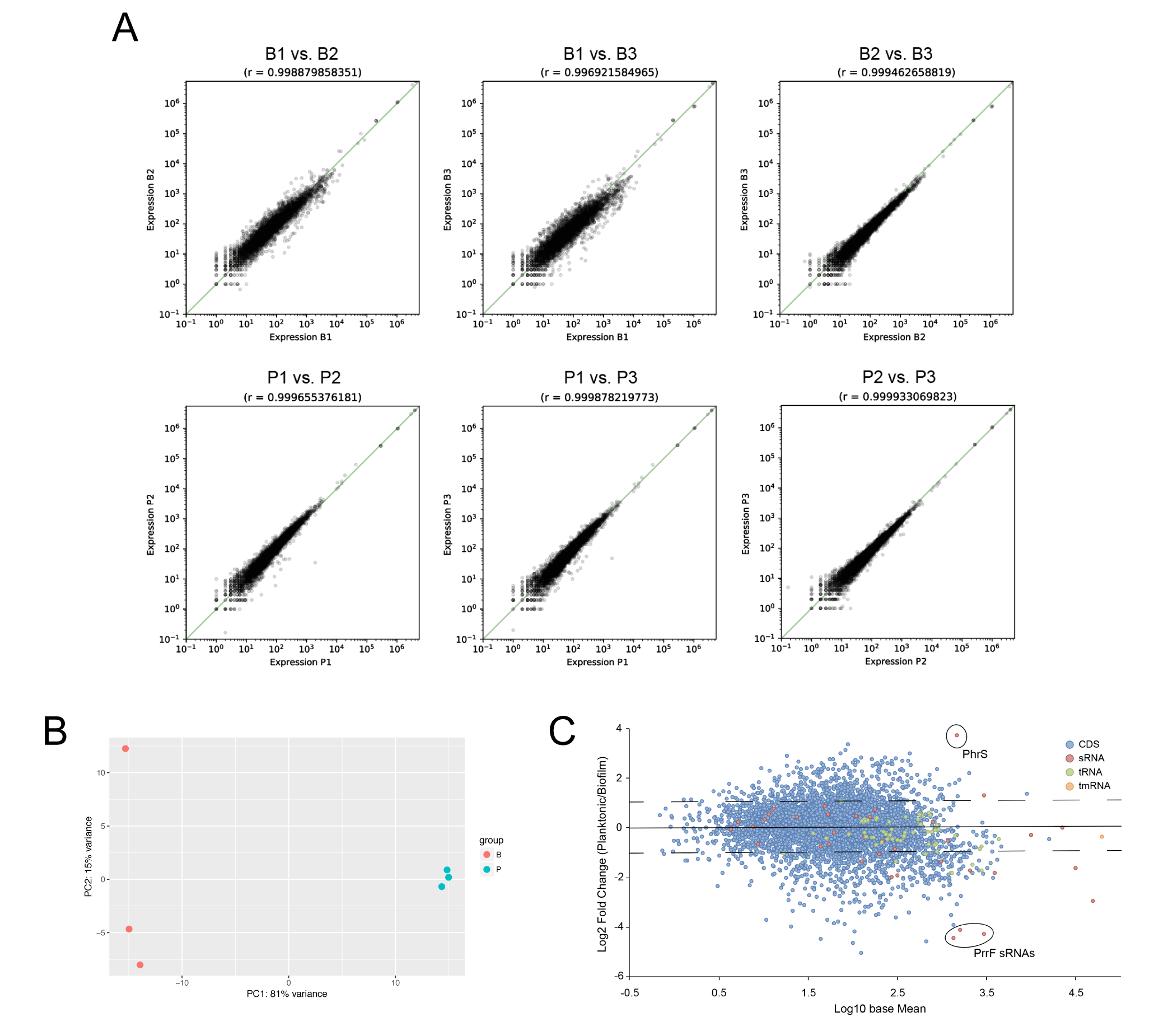

Supplement: FIG S6 [file mSystems.00590-19-sf006.tif]
