# Supplementary material for: Conditional Hfq Association with Small Noncoding RNAs in Pseudomonas aeruginosa Revealed through Comparative UV Cross-Linking Immunoprecipitation Followed by High-Throughput Sequencing
Source: mSystems. 2019 Dec 3;4(6):e00590-19. doi: 10.1128/mSystems.00590-19 (PMC6890931; doi:10.1128/mSystems.00590-19)
Supplement: TABLE S4 [file mSystems.00590-19-st004.docx]

**Table S4. The list of strains, plasmids, and oligonucleotides.**

| **Strains** | | | | | | | | | | |
| --- | --- | --- | --- | --- | --- | --- | --- | --- | --- | --- |
| **No.** | **Name** | **Specie** | **Strain** | | **Genome edit** | **Description of strain** | | | **Source** | |
| 1 | KCS-0015 | *P. aeruginosa* | PAO1 | | Null | *Pseudomonas aeruginosa* PAO1 Wild Type. | | | Jörg Vogel, Univ. Würzburg | |
| 2 | KCS-0016 | *P. aeruginosa* | PAO1 | | *hfq*::3xFLAG | 3xFLAG was cloned into downstream of genomic *hfq* | | | This study | |
| 3 | KCS-0019 | *P. aeruginosa* | PAO1 | | Δ*hfq* | *hfq* was removed from PAO1 genome | | | This study | |
| 7 | KCS-0017 | *E. coli* | S17-1 | | Null | *recA pro hsdR* RP42Tc::MuKm::Tn7 integrated into the chromosome | | | Simon, R. et al., Methods Enzymol, 1986 (1) | |
| 8 |  | *E. coli* | DH5α | | Null | F-, Φ80d*lacZ*ΔM15, Δ(*lacZYA-argF,* U169, *deoR*, *recA*1, *endA*1, *hsdR*17(r_K_-, m_K_+), *phoA*, *supE*44, λ-, *thi*-1, *gyrA*96, *relA*1 | | | TAKARA | |
| **Plasmids** | | | | | | | | | | |
| **No.** | | **Plasmid name** | | | | **Origin / marker** | | **Reference / Source** | | |
| 1 | | pSUB11 | | | | R6K / AmpR | | Uzzau, S. et al., PNAS, 2001 (2) | | |
| 2 | | pG19II | | | | pMB / GmR | | Maseda, H. et al., AAC, 2004 (3) | | |
| 3 | | pG19UFD | | | | pMB / GmR | | This study | | |
| 4 | | pG19Δhfq | | | | pMB / GmR | | This study | | |
| **Oligonucleotides** | | | | | | | | | | |
| **No.** | **Oligo number** | **Name** | | **Sequence** | | | **Description of oligo** | | | **Reference** |
| 1 | KCO-0001 | hfqUP_f | | GGGaagcttGTTTCCAGCTGGGTCTCGCCC | | | Cloning of 500 bp upstream fragment forward, contains HindIII | | | This study |
| 2 | KCO-0002 | hfqUP_r | | GGGctgcagCGGGAGTCCGCTTTGTTCTTTGAG | | | Cloning of 500 bp upstream fragment reverse, contains PstI | | | This study |
| 3 | KCO-0003 | hfqDWN_f | | GGGctgcagGTGCCGCACTCCTTTAAGGATCGTA | | | Cloning of 500 bp downstream fragment forward, contains PstI | | | This study |
| 4 | KCO-0004 | hfqDWN_r | | GGGtctagaCGTCAGGCAGGAATCAATTGCCG | | | Cloning of 500 bp downstream fragment reverse, contains XbaI | | | This study |
| 5 | KCO-0005 | hfqFLAGUP_f | | GGGAAGCTTtgtgcggctcgaccgagg | | | Cloning of 1kb downstream fragment forward, contains HindIII site | | | This study |
| 6 | KCO-0006 | hfqFLAGUP_r | | caaagatgacgacgataaatagtaacgggagtccgctttgttctttga | | | Cloning of 1kb downstream fragment reverse, overlaps 3x FLAG tag | | | This study |
| 7 | KCO-0007 | FLAGx3_f | | gctcaaagaacaaagcggactcccgttactatttatcgtcgtcatctttgtagtc | | | Cloning of 3x FLAG tag forward, overlaps downstream fragment | | | This study |
| 8 | KCO-0008 | FLAGx2_r | | ccagccggccgagccgggcaacgctgactacaaagaccatgacggtgatt | | | Cloning of 3x FLAG tag reverse, overlaps upstream fragment | | | This study |
| 9 | KCO-0009 | hfqFLAGDWN_f | | aatcaccgtcatggtctttgtagtcagcgttgcccggctcgg | | | Cloning of 1kb upstream fragment forward, overlaps 3x FLAG tag | | | This study |
| 10 | KCO-0010 | hfqFLAGDWN_r | | GGGGGATCCggcgttgctcgagggcct | | | Cloning of 1kb upstream fragment reverse, contains BamHI site | | | This study |

1. Simon R, O'Connell M, Labes M, Puhler A. 1986. Plasmid vectors for the genetic analysis and manipulation of rhizobia and other gram-negative bacteria. Methods Enzymol 118:640–59. doi:10.1016/0076-6879(86)18106-7

2. Uzzau S, Figueroa-Bossi N, Rubino S, Bossi L. 2001. Epitope tagging of chromosomal genes in Salmonella. Proc Natl Acad Sci U S A 98:15264–15269. doi:10.1073/pnas.261348198.

3. Maseda H, Sawada I, Saito K, Uchiyama H, Nakae T, Nomura N. 2004. Enhancement of the mexAB-oprM Efflux Pump Expression by a Quorum-Sensing Autoinducer and Its Cancellation by a Regulator, MexT, of the mexEF-oprN Efflux Pump Operon in Pseudomonas aeruginosa. Antimicrob Agents Chemother 48:1320–1328. doi:10.1128/aac.48.4.1320-1328.2004.
